# Oncoplastic surgery in the treatment of breast cancer

**DOI:** 10.3332/ecancer.2013.293

**Published:** 2013-02-21

**Authors:** Alberto Rancati, Eduardo Gonzalez, Julio Dorr, Claudio Angrigiani

**Affiliations:** University of Buenos Aires, Buenos Aires, Argentina; ^1^ This article is a general review based on: González E, Morgado CC, Noblía C, Armanasco E, Azar M, Montoya D and Ipiña M 2006 **Utilidad y Sistematización de las Técnicas de Cirugía Oncoplástica en la Prevención y Corrección de las Secuelas del Tratamiento Conservador Consideraciones Oncológicas y Cosméticas ***Revista de la Sociedad Argentina di Mastologia ***25** 86

## Abstract

Advances in reconstructive breast surgery with new materials and techniques now allow us to offer our patients the best possible cosmetic results without the risks associated with oncological control of the disease. These advances, in both oncological and plastic surgery, have led to a new specialisation, namely oncoplastic breast surgery, which enables us to undertake large resections and, with advance planning, to prevent subsequent deformities. This is particularly important when more than 30% of the breast volume is removed, as it allows us to obtain precise information for conservative surgery according to the site of the lesion, and also allows us to set the boundary between conservative surgery and mastectomy.

Given the existence of new alloplastic materials and new reconstructive techniques, it is essential for our patients that surgeons involved in breast cancer treatment are trained in both the oncological as well as the reconstructive and aesthetic fields, to enable them to provide the best loco-regional treatment with the best cosmetic results.

## Definition and concept of oncoplastic surgery in the treatment of breast cancer

A great deal has been written in recent years on the topic of oncoplastic breast surgery (OBS), probably without considering the original definition of the term. According to the writings of Audretsch [1] OBS originally included all approaches of plastic and reconstructive surgery aimed at achieving tumour resections with satisfactory margins in conservative treatment, thereby attempting to minimise potential deformities and to obtain the best possible cosmetic results.

The technique had been given several names such as cosmetic quadrantectomy by Silverstein [2], low pole tumour reduction mammaplasty by Clough [3], and central tumour reduction by Grisotti [4], etc., but subsequently the concept was accorded the term tumour specific immediate reconstruction [5]. Proposed by the US plastic surgeon John Bostwick III in 1996, the term includes not only techniques preventing the consequences of conservative treatment but also a whole range of techniques involving partial or total immediate post-mastectomy reconstruction (immediate breast reconstruction), correction of their consequences (delayed breast reconstruction), and immediate repair of the surgical treatment of locally advanced tumours and recurrences in the chest wall. Nowadays, following a period of uncertainty in the nomenclature, the term OBS is uniformly associated in the medical community with the classification system of John Bostwick III, in both Latin and Anglo-Saxon publications.

It is important to clarify that the term OBS also encompasses the techniques developed for preventive surgery in high-risk patients (risk reduction mastectomies).

## Classification

OBS may be classified schematically into:

(a) Post-mastectomy breast reconstruction (PMBR)

1. Immediate breast reconstruction (PM-IBR). This is done when the tumour is resected.
  Depending on whether the skin of the breast or the nipple-areola complex (NAC) is resected, IBR may be classified in turn as:
  (i) Conventional or no skin sparing mastectomy (No-SSM), when the cutaneous layer is resected without leaving excess breast skin behind.
  (ii) Skin sparing mastectomy (SSM), when as much of the cutaneous layer as possible and the sub-mammary fold are preserved, but with resection of the NAC and prior biopsy incisions and/or diagnostic percutaneous biopsy scarring.
    SSM may in turn be subdivided into five groups [6–8]:
    (A) Periareolar or lozenge resection of the NAC, sparing the skin of the breast;
    (B) Resection of the NAC with a medial or lateral extension resecting previous biopsy scarring;
    (C) Periareolar resection of the NAC and separate incision resecting previous biopsy scarring;
    (D) Border lozenge resection of skin including the NAC, in an attempt to reduce ptosis (indicated for ptotic and hypertrophic breasts);
    (E) Resection of the skin and NAC in an inverted T shape (ptotic and hypertrophic breasts).
  (iii) Skin and areola sparing mastectomy (ASM), when the entire cutaneous cover, areola, and sub-mammary fold are preserved, but the nipple and incisions from previous biopsies and/or scarring from diagnostic percutaneous biopsies are resected.
  (iv) Nipple sparing mastectomy (NSM), when the entire cutaneous layer, areola, nipple, and sub-mammary fold are preserved, as well as incisions from previous biopsies and/or scarring from diagnostic percutaneous biopsies.

2. Delayed breast reconstruction (PM-DBR). The period of time in which this is done varies after the mastectomy.

(b) Conservative post-surgery reconstruction
  3. Immediate breast reconstruction (CS-IBR). This is done at the time of the partial tumour resection from the breast to prevent the consequences of resection and of subsequent radiotherapy (RT).
  4. Delayed breast reconstruction (CS-DBR). The period of time in which this is done varies after the conservative treatment to correct the consequences of surgery and radiotherapy.
(c) Reconstruction of defects in the chest wall and in soft tissues, secondary to surgical treatment of locally advanced breast cancer and of extensive local recurrences.

## Post-mastectomy breast reconstruction (PMBR)

## Oncological and technical considerations (ODF)

In PMBR, each patient needs to be assessed and her treatment based on the following factors:

- factors likely to increase morbidity;
- factors allowing the time of reconstruction and technique to be determined.

### Factors likely to increase morbidity

Various factors may influence the decision on breast reconstruction, according to the number of complications, and hence selection of the technique and time, such as:

- Obesity
- Diabetes
- Smoking status
- Autoimmune diseases
- Previous radiotherapy

Essentially, obesity, smoking, and previous radiotherapy may cause an increase in the number of complications involving any of the current reconstruction techniques most frequently cited. Obesity can increase complication rates [9] by up to 12 times, and smoking may directly affect the vasoconstrictor effect in the skin and indirectly inhibit capillary flow owing to the release of nicotine-mediated catecholamines, producing increased necrosis in skin flaps resulting from the mastectomy [10, 11] and various vascular disorders in techniques involving autologous tissue [12, 13]. The administration of radiotherapy may alter the reconstruction process owing to potential vascular alteration of the foundation tissue or the occurrence of some degree of fibrosis associated with scarring disorders [15, 16].

Although as an individual factor diabetes does not significantly increase complications, its impact is the greatest when obesity and/or radiotherapy is/are involved, leading to an increase in the number of infections and cutaneous necrosis [12–14].

Despite the paucity of literature on the subject, patients with autoimmune conditions may usually undergo reconstruction if their condition is stable, but it is better to avoid techniques, which use prostheses because of their association with antinuclear antibodies (ANA) and the development of anticardiolipin syndrome [17, 18].

## Factors enabling the time of reconstruction and technique to be determined

The following factors must be evaluated here:

### Immediate or delayed breast reconstruction?

IBR is currently indicated for the majority of patients at centres providing training in oncological and reconstructive treatment for breast cancer. However, there are a number of issues to resolve here, such as: on the one hand, the suitability of this procedure, the selected technique, the possibility of providing ancillary radiotherapy and its consequences, and on the other, the long-term cosmetic and psychological outcome and impact on patients’ quality of life.

In the United States, there are publications stating that, despite the oncological safety and aesthetic benefit provided by IBR, it is undergone by fewer than 20% of patients [19]. Socioeconomic, ethnic, geographical, and educational factors play a significant role in opting for immediate procedures [20].

### Oncological decision-making factors (ODF)

In IBR, ODF need to be taken into account, as these factors influence the time selected for reconstruction because of possible clashes between the reconstructive procedure and ancillary treatments and their effect on the complication rate.

“Technical considerations” that directly influence the final cosmetic result in IBR also require consideration.

ODFs involve:

- Histological features of the tumour, size, growth speed, etc. (a histological biopsy should preferably be obtained by puncture, core or mammotome);
- Evaluation of the axiliary condition clinically or by additional noninvasive methods (echogram, PET), minimally invasive methods (cytology, core biopsy), or a deferred biopsy of the sentinel ganglion under local anaesthetic.

The importance of evaluating these decision-making factors is directly related to the need for ancillary post-mastectomy radiotherapy treatment and its implications for selecting the time (IBR or DBR) or technique (implants or autologous tissue) of reconstruction.

It must be emphasised that all elements used in assessing ODFs should involve the judgement and experience of the professionals concerned.

In light of the above, we may examine two particular situations:

### Sentinel Node (SN) technique and immediate breast reconstruction

The inclusion of an investigation of the sentinel ganglion in breast cancer treatment and the decision whether to act or not according to the result of the intra-operative biopsy has made it necessary, when IBR is indicated, to examine carefully the reconstruction technique to be adopted.

If we opt for prostheses or implants, any need for axiliary drainage subsequent to a false negative in the frozen-section biopsy is not problematic, since with an axiliary incision independent of the mastectomy scar the objective can be attained without altering the outcome of reconstruction.

When we indicate an IBR with a microsurgical flap of abdominal tissue, venous, and arterial anastomosis is routinely achieved with internal mammary vessels to avoid damaging an anastomosis in the area concerned (thoracodorsal vessels), should an axiliary re-exploration be necessary.

### Immediate breast reconstruction and ancillary treatments

It has been published and demonstrated that IBR supported [21, 22] by chemotherapy (CT) does not delay the start of treatment or increase the number of complications involved in reconstruction.

The situation is not so clear when post-mastectomy ancillary radiotherapy (PMRT) is necessary.

The requirements for PMRT have changed in recent years and it should be considered for patients on the basis of their ganglial condition, tumour size and other prognostic histological factors. Axiliary condition, irrespective of tumour size, with axillae registering positive with one to three or four or more ganglia compromised, may indicate a need for PMRT indiscriminately, according to experience or current treatment protocols [23].

These guidelines are all the more important when selecting IBR in view of the potential interaction of implants (the technique most often recommended currently for IBR) with radiotherapy [24]. Therefore, interdisciplinary collaboration is essential. Support and reconstruction times and the type of reconstruction to be used must be considered by the surgeon, oncologist, and radiotherapist together.

It is known that reconstruction using implants and prostheses is most commonly employed nowadays (76% of all breast reconstructions in the USA at the American Society of Plastic Surgery, 2007), and the adverse effects of radiotherapy on the cosmetic results of such reconstructions in terms of increased complications and the high rate of severe capsular contractures (around 50%) are also known [25, 26].

On the other hand, the use of implants with an incorporated metal valve is generating controversy in view of the technical difficulties that this valve can cause for the proper application of radiotherapy and the homogeneity of administered doses [24–27].

These alternatives mean that we need to examine the indications of IBR and divide patients into two clear groups. On the one hand, those who will very likely require PMRT and those whose BR should be deferred or subject to discussion with the patient, who should be offered a reconstructive technique that better tolerates PMRT than implants and prostheses. In these specific situations, BR with autologous tissue (tram flap and all its varieties: pediculate, microsurgical, etc.) may be indicated. Although these techniques tolerate radiotherapy better than prosthetic materials, they may have fibrous effects and unsatisfactory results over time, with secondary corrections being required in around 25% of cases [28]. Despite the controversy in the literature, we should mention a recent meta-analysis by Barry [29] who analysed 1105 patients from 11 suitably selected studies, who had undergone BR and PMRT, concluded that when supporting radiotherapy was necessary BR with autologous tissue generated less morbidity and better results over time in comparison with prosthesis-based BR.

The second group contains patients not needing PMRT and who can be safely offered IBR with implants and/or prostheses.

Special mention should be given to the situation in which PMRT is indicated after the insertion of a breast implant with incorporated metal valve. In that case, the strategy to be followed, based on the experience of Memorial Sloan Kettering of New York [30], is first to carry out supportive chemotherapy, exchange the implant for a silicone prosthesis, and then administer radiotherapy. This sequence avoids the need to irradiate the implant, with the problems that this may entail, and achieves a more harmonious result whilst reducing the number of complications. Arecent publication by Maurizio Nava of the National Tumour Institute in Milan, Italy [31] demonstrates that this delay in carrying out RT to position the prosthesis permanently before it is administered reduces the number of complications and does not increase the rate of local recurrences, compared with irradiating the implant before its replacement. Another published alternative, perhaps more complex and with more secondary complications, is that proposed by Kronowitz of the MD Anderson Cancer Centre in Houston, Texas [32] whereby the tissue implant is deflated before administering PMRT and is then replaced with a breast reconstruction involving microsurgical autologous tissue.

## Technical considerations influencing the IBR procedure and aesthetic result

### Skin and sub-mammary furrow sparing mastectomies

In 1991 Toth and Lappert [33] were the first to use the term SSM, which they defined as a modified mastectomy procedure, either simple or radical, involving incisions that limit its resection, including the nipple-areola structure, previous biopsy scarring or the skin near a superficial tumour, allowing access to the axilla for possible drainage, and potentially associated with an additional axiliary incision, if necessary, to facilitate the reconstructive procedure selected.

Use of these techniques has not shown an increase in the rate of local relapses compared with conventional mastectomies without reconstruction [34].

Local relapses, therefore, represent a consequence of tumour biology rather than a problem with the surgical technique, and hence not extending the limit of skin resection does not appear to alter the relapse rate [35].

## Mastectomies with preservation of the skin and NAC

Between 1975 and 1985, many reports of attempts to preserve the NAC in mastectomies were published. This technique was quickly abandoned owing to the high incidence of invasion of the nipple base observed in all studies (between 8% and 50%) [36].

In 1999, C. Laronga [37] published a new report on this technique, recording low rates of nipple compromise in patients with selected breast tumours (small tumours, well differentiated, distant from the complex, and with negative axilla), thereby reintroducing the possibility of including this procedure into current breast cancer treatment. Further publications confirm the findings of other authors [38, 39].

In 2008, the Veronesi and Petit group at the European Oncological Institute in Milan [40] published a study of this technique involving the preservation of the NAC but with the inclusion of intraoperative radiotherapy (ELIOT), with good oncological and cosmetic results but a short monitoring period.

The oncological safety of preserving the NAC has not yet been confirmed by prospective randomised studies. To date, a number of retrospective studies have not observed a local recurrence rate higher than that expected when comparing with conventional mastectomies and skin-sparing procedures [41].

As stated by the National Comprehensive Cancer Network (NCCN) in its 2012 version [23], in all these procedures (SSM-NSM) it is essential that the surgical team is experienced in the technique, patients are correctly selected (stage, ancillary treatment, etc.), previous disease inducing factors (smoking, radiotherapy) are correctly evaluated and suitable resection margins are obtained.

Indications of therapeutic NSMs must still be taken into account in investigation protocols [23].

### NSM indications, investigation protocols

- Distance of the tumour from the NAC >2 cm (determined by mammogram or NMR);
- Tumour <o = 2.5 cm (determined by clinical procedure, echogram, mammogram or NMR);
- No vascular lymphatic invasion;
- Axiliary ganglia clinically negative;
- Sub-group of patients recommended for conservative treatment and preferring a mastectomy;
- Risk-reducing mastectomies.

## Resection techniques and immediate breast reconstruction in risk reduction mastectomies

The name of these procedures is due to the benefits of risk reduction in specific high-risk groups (patients carrying BRCA I and II mutations, previous family history of breast cancer, breasts that are difficult to monitor, treated breast cancer with associated high-risk factors, etc.) [42]. They are inaptly known as ‘prophylactic’.

This reduction benefit is noted in between 90% and 95% of patients, and so the term “prophylactic” is not appropriate here, given the evidence that in patients with risk reduction mastectomies between one percent and 10% may develop a breast carcinoma in residual breast tissue [43, 44].

It is as technically valid to resect the NAC as it is to preserve it. Investigation of the sentinel ganglion, enables an evaluation of possible hidden lesions within it. Also if it is intact, completion of the surgical procedure in situations of neoplastic pathology in the mastectomy area detected in the deferred biopsy, is currently under discussion. There are disagreements as to whether it should be performed always, never, or only in selected cases (e.g. older patients or those with a history of lobulillar carcinoma or CLIS) [45–47].

As for reconstruction, we can say that in most cases silicone prostheses, implants, and rarely, miocutaneous flaps are recommended.

To summarise: in most of the literature on IBR it has been shown that:

- It does not interfere with or obstruct exploration or treatment of the axilla (axiliary drainage, SG) in primary surgery or if a secondary procedure is necessary in case of a false negative in the frozen-section biopsy of the SG;
- It does not obstruct or delay the implementation of ancillary chemical treatments, whether CT, hormonotherapy (HT), trastuzumab, etc. [48];
- It may obstruct correct administration of PMRT, depending on the reconstructive technique selected (e.g. incorporated-valve implants), and although the subject is controversial there are alternative ways of overcoming this problem (exchanging an implant for a prosthesis before starting radiotherapy);
- It does not interfere with monitoring of the reconstructed breast (see item Image Monitoring of the Reconstructed Breast) [49];
- It slightly increases the number of complications compared with deferred breast reconstruction, but its impact on aesthetic outcome is no greater for immediate breast reconstruction and does not affect the time, or form of ancillary treatments to be carried out [19–50];
- Sparing or not sparing skin does not increase or interfere with the treatment of local relapses, with the incidence of resecting reconstructed breasts due to relapses being approximately one percent [51, 52].

## Indications of immediate breast reconstruction

- Patients recommended for a mastectomy due to stage I and II breast cancers;
- Patients recommended for a mastectomy in cases of local recurrences after conservative treatment, without indications of an acute carcinoma;
- Patients recommended for a mastectomy in some selected stage III cases (T3 N1), with a good response to induction treatments [53];
- In all these instances, it is possible to preserve the skin of the breast (SSM), provided that the oncological margins of the resection are not compromised and account is taken of factors which may increase complications such as previous scarring, previous radiotherapy, or a history of smoking;
- For recommendations of IBR due to local recurrences after conservative treatment or cases at stage III (previously irradiated patients) it is advisable to use reconstructive techniques based on autologous tissue (Tram and its varieties) in order to reduce the number of complications and to improve cosmetic results [54]. It is not advisable to employ IBR with prosthetic materials in these cases [55];
- Patients with locally advanced tumours with or without the chest wall affected, where the immediate reconstructive procedure is needed to repair defects in costal, sternal or soft-tissue resections;
- Patients recommended for risk-reducing mastectomies.

## Contraindications of immediate breast reconstruction

- Patient is opposed to reconstruction [56];
- Psychological conditions evaluated and diagnosed by a specialist, such as serious mental health problems which prevent understanding of the procedure, suspected dysmorphophobic syndrome, etc.;
- Impossibility or uncertainty of achieving oncologically adequate margins after applying primary systemic therapy;
- Possibility of ancillary radiotherapy, which makes it difficult to perform IBR or affects the cosmetic outcome (relative contraindication);
- Accompanying illnesses which make performance of the reconstructive technique risky.

### Deferred breast reconstruction

In patients suffering from the after effects of a mastectomy it is feasible to carry out reconstruction at a later date, once supporting treatment is complete and the patient has recovered from any illnesses. There is currently no fixed waiting time between the end of treatment and the time of reconstruction.

In this situation, the negative impact of surgery, presence of the pectoral muscles, intact nature of the thoracodorsal vessels, skin quality, presence or absence of effects due to previous radiotherapy and possible tissue donor areas in case of necessary skin-flap or lipo-transfer techniques must be assessed.

In recent years, there has been a limited and highly selective literature published on DBR in patients with stage IV breast cancer; e.g. bone progression medicated, controlled and stable and in a generally good condition. In such cases, common sense should prevail and nonaggressive low-morbidity reconstructive techniques should be recommended [57].
